# PrEP awareness and decision-making for Latino MSM in San Antonio, Texas

**DOI:** 10.1371/journal.pone.0184014

**Published:** 2017-09-27

**Authors:** Moctezuma García, Allyssa L. Harris

**Affiliations:** 1 School of Social Work, Texas State University, San Marcos, TX, United States of America; 2 Connell School of Nursing, Boston College, Chestnut Hill, MA, United States of America; David Geffen School of Medicine at UCLA, UNITED STATES

## Abstract

Pre-Exposure Prophylaxis (PrEP) has been promoted among high-risk populations as an effective HIV biomedical intervention. However, limited research is available on the significance of culturally informed biomedical interventions for Latino MSM. A total of 159 self-administered Internet surveys were completed by Latino MSM ages 21–30 in San Antonio, Texas. The purpose of this research was to develop an instrument that measured Latino MSM attitudes and beliefs towards PrEP, identify associations between demographic factors and PrEP related factors and to suggest culturally appropriate strategies for the promotion of PrEP among the Latino MSM population. Research findings revealed implications for PrEP at the structural and individual level for Latino MSM. Structural level indicators emphasized the importance for raising PrEP awareness among Latino MSM in regards to PrEP related expenses, ameliorating stigmatization of high-risk populations, enhancing access to PrEP informed medical providers, and address mistrust of the government and medical providers role on addressing health disparities among Latino MSM. Overall, the findings for individual factors emphasize the need for patient-centered interventions for Latino MSM. Latino MSM currently on PrEP require supplemental resources to enhance PrEP adherence. Latino MSM not on PrEP require alternate options for PrEP delivery and/or cognitive behavioral approaches minimizing HIV risk behavior for Latino MSM concerned with PrEP toxicity, which may require non-biomedical interventions. Integration of Latino MSM currently on PrEP as peer educators provides a valuable resource for developing culturally informed PrEP interventions for Latino MSM. Peer educators are able to share their experiential knowledge of PrEP contextualized through cultural norms, beliefs, and values.

## Introduction

In 2012 the United States Food and Drug Administration approved Truvada as a Pre-Exposure Prophylaxis (PrEP) biomedical intervention to prevent the transmission of HIV among high-risk populations that are disproportionately affected by HIV, which include Men who have Sex with Men (MSM) [1]. The PrEP medication regimen requires individuals to take a pill daily and HIV transmission is reduced by 92% with medication adherence [2]. In addition, individuals are also advised to meet with a medical provider every three months to assess for medication side effects and monitoring of their HIV status. In an effort to meet U.S. National HIV/AIDS Strategy goals to reduce HIV transmission in designated HIV burdened cities, the Centers for Disease Control and Prevention (CDC) has provided financial support to states for the development and implementation of PrEP programs [3]. However, research has demonstrated that many high-risk populations are unaware of PrEP as an effective strategy for the reduction of HIV transmission [4–6]. This is especially true among the MSM population where only 57% of MSM are aware of PrEP [7]. MSM in general have been reluctant to consider PrEP for multiple reasons including the required daily pill regimen [8, 9], costs associated with PrEP [8–15], and the medication’s long-term health effects [8–11, 13–19]. MSM of color (e.g., Latino, Black) in particular have expressed a lack of trust of the medical community [16, 19–22], which hinders efforts in promoting PrEP among populations at greatest risk of HIV exposure. Additionally, health care providers also express concerns about patient medication adherence, side effects, and continued engagement in high-risk behaviors among MSM [7, 9, 23].

The literature was reviewed to develop a survey that assesses PrEP related attitudes and beliefs for Latino MSM. Three significant limitations were noted. First, there was a lack of culturally appropriate quantitative instruments that measured PrEP related attitudes and beliefs for Latino MSM. Secondly, no PrEP instruments were found that measured PrEP attitudes among Latino MSM from the southern portion of the U.S. Finally, no PrEP instrument in the Spanish language were found; this significantly limits the applicability of PrEP interventions for the Latino population. In addition, multiple qualitative studies reported that MSM of color do not trust the government [16, 20, 21]. However, there is a dearth of quantitative research exploring the role of government and state agencies in providing PrEP as a biomedical HIV intervention for MSM of Color including Latino MSM. Many current instruments also do not include questions about potential PrEP related adverse side effects, medication adherence, and adherence to follow-up medical appointments [9]. All of which are important decision-making factors for MSM to consider PrEP. To date there are few studies that focus on Latino MSM and culturally appropriate PrEP biomedical interventions.

### Background

PrEP as a biomedical intervention has raised prominent concerns that MSM taking PrEP would be more likely to forgo condom use [9, 10, 24] and have an increased number of multiple sex partners [13, 24]. One possible explanation for elevated risk behavior (e.g., condomless sex, multiple sex partners) as a result of PrEP use is risk compensation theory, which indicates that an individual modifies one’s behavior based on perceived risk [10, 25, 26]. Research indicates that PrEP users (e.g., males, females, heterosexual, MSM) may gain a sense of safety from not being susceptible to HIV infection, which potentially may encourage increased engagement in risky behaviors [27]. Therefore, MSM PrEP users may modify their behavior and partake in condomless sex due to a perceived diminished risk of HIV transmission, also known as “PrEP Optimism” [10].

Qualitative research participants (39 MSM, including 20 Latino MSM) expressed concerns that PrEP use may result in greater condomless sex, but the results indicated that there was no change in condom use before and during PrEP use [13]. Medical providers also indicated in a qualitative study that PrEP patients reported an increase as well as decrease in risky behaviors [28]. Quantitative research studies in contrast have demonstrated higher rates of condomless sex [10, 24, 29]. A New York City PrEP study with 152 HIV negative MSM (26.3% Latino) found that MSM PrEP users were four times more likely to have condomless sex [10]. Data based on the National HIV Behavioral Surveillance (NHBS) from 2004 to 2014 (n = 1211 for 2004–19.1% Latino; n = 407 for 2008–24% Latino; n = 371 for 2011–20.2% Latino; and n = 301 for 2014–25.4 Latino) indicated that the introduction of PrEP was associated with greater levels of condomless sex, multiple sex partners, and sexually transmitted infections (STIs) for MSM in San Francisco [24]. Another study found an increase of PrEP users (N = 972, 97.9% male—12.2% Latino) with an STI from 15.9% at baseline to 32% in the previous two years [29], which is an important indicator of condomless sex. The aforementioned research indicates a need for research endeavors to explore PrEP Optimism and condomless sex as a decision-making factor for high-risk populations using PrEP.

In addition to concerns around the increase of condomless sex PrEP medication adherence has been reported as a significant problem. PrEP efficacy ensures effectiveness, which requires one to take a minimum of 4 doses per week [29]. However, medication possession ratio indicates that patients must have a minimum of 80% and anything below requires supplemental support to enhance PrEP adherence [29]. Findings from a randomized control trial with 50 MSM (8% Latino MSM) who were PrEP naïve and participated in condomless sex had higher levels of Tenofovir, which indicated PrEP adherence, when they received cognitive-behavioral therapy compared to the control group [30]. However, findings from the same study [30] indicate that MSM with current mental health and substance use issues were less likely to engage in PrEP medication adherence [31]. Lower PrEP adherence among 972 MSM (12.2% Latino) in Northern California was associated with higher copayments for PrEP and smoking, but discontinuation of PrEP was greater among MSM reporting substance abuse [29]. Research findings indicate that PrEP as a biomedical intervention requires supplemental interventions tailored to addressing mental health as well as substance use to strengthen PrEP adherence for MSM.

Research has also explored other avenues of PrEP medication administration and adherence. “On-demand” PrEP is an effort to enhance medication adherence that requires an individual to take 2 pills 24 hours prior to sex, 1 pill 24 hours after sex, and 1 pill 48 hours after sex [32]. Study results indicate that “on-demand” PrEP was 86% effective in reducing HIV infection and was considered to be an effective alternative among high-risk MSM (N = 414, 95% White–Latino not specified) that do not use condoms consistently [32]. However, based on results from a national survey highly sexually active MSM (N = 948–12.6% Latino) are unlikely to predict when they will have sex, which limits the efficacy of “on-demand” PrEP [16]. A long-acting injectable PrEP regimen (1 injection every 3 months) is currently being explored in clinical trials and 51.3% of Latino MSM (n = 119) reported that they prefer an injection of PrEP [16]. However, 18.5% of Latino MSM indicated no preference between the current pill form versus the long-acting injectable form of PrEP [16].

Researchers have also noted that MSM experience seasonal sexual risk trajectories which takes into account HIV related risk behaviors associated with age, distress or depression, and substance use during a particular time period [33–35]. A large U.S. based internet study of MSM (N = 7,305–7.5% Latino) found that 92.6% of MSM reported that taking PrEP daily was a barrier [35]. However, 74.3% of MSM would be willing to take PrEP during short periods of risk. MSM are also more likely to plan vacation destinations based on sex [35, 36]. This vacation pattern likely indicated an increased engagement in risky behaviors such as condomless anal sex and multiple sex partners [35, 36]. The use of “on demand” PrEP during seasonal risk trajectories may be an effective tool in reducing HIV transmission. Overall research has demonstrated a need for the development of patient-centered HIV biomedical and behavioral interventions for Latino MSM. However, limited research and social services among this population has created structural, psychological, social, and geographical barriers related to PrEP access.

Research demonstrates that PrEP for high-risk populations is cost effective for high HIV burdened areas; but considerations must be taken related to drug expenses and PrEP related service costs (e.g., HIV testing, laboratory tests, PrEP related adverse outcomes) [37, 38]. However, the intersections of race, sexuality, HIV status, political ideology, and geographical area influence health disparities and access to care for MSM of color. Structural factors (e.g., access to health insurance, transportation, socioeconomic conditions) supplemented with stigmatizing attitudes against people of color, People Living with HIV (PLWH), and the gay community have resulted in very low levels of PrEP awareness, access, and acceptance for Black MSM in the South [39]. A national study explored PrEP related attitudes among the general public (N = 154–87.7% heterosexual, 43.8% male, 78.6% White, and 11% Latino) and found that tailored PrEP campaigns for Black MSM reinforce stigmas that Black MSM are sexually promiscuous and prone to health care disparities [40]. Research found significant racial and sexual PrEP funding disparities when public funding initiatives specifically target Black MSM [40]. Therefore, the research emphasizes the need to explore additional frameworks to raise PrEP awareness as a benefit for society and mitigate stigmatization of highly marginalized populations [40].

Despite the mainstreaming of HIV care, significant barriers remain for HIV testing and treatment by health care providers. According to research only 35% of primary care providers offer routine HIV testing for their patients, but 81% are willing to provide HIV testing upon patient request [41]. Eighty-three percent of primary care providers are willing to engage in continuing medical education on PrEP, but 59% indicate that they should be reimbursed (range of $50-$100) for PrEP counseling [41]. A significant barrier among primary care providers include a lack of knowledge about PrEP compared to HIV specialty providers [7, 14, 23]. Hence, primary care providers are less likely to prescribe PrEP due to concerns related to medical side effects, adherence, and/or sexual risk behaviors [7, 23, 28]. Primary care providers may also have an increase in racial bias when considering whether a patient is appropriate for PrEP [42]. Research indicates that Black MSM are less likely to receive a PrEP prescription due to a medical provider’s racial bias [42]. MSM of color (N = 491–23% Latino and 33% Black) are also more likely to utilize public health services, report PrEP related stigma, and avoid disclosing one’s sex life with their doctor [43]. MSM of color encounter unique systemic barriers associated with racism and homophobia from health care providers, which hinders efforts in increasing PrEP awareness and preventing MSM of color from making informed decisions [39, 43].

Improving health care providers’ knowledge of PrEP enhances patient-centered counseling on PrEP adherence, potential side effects, and the importance of long-term monitoring [13, 14]. Results from a recent study of MSM participants (n = 38 in focus groups– 11% Latino; n = 56 individual interviews– 16% Latino) found that individuals’ expressed anxiety around the interactions of PrEP and current medication regimens as well as with co-existing medical conditions [44]. Appropriately addressing PrEP related anxieties is dependent on the medical provider’s knowledge of PrEP. However, in a qualitative study of 14 MSM (7% Latino) participants reported a fear of being judged when requesting PrEP despite reporting a close and trusting relationship with their medical provider [45]. MSM report that a health care provider’s enhanced understanding of PrEP, holding nonjudgmental sex-positive communication as well as offering a nonjudgmental safe space which promotes open communication is important for on-going health maintenance. Furthermore, MSM have reported being able to distinguish the difference between educating (e.g., telling a patient what to do) versus understanding a patients needs for treatment [45]. A patient-centered approach requires a health care provider to advise the patient on making an informed decision regarding the risks and benefits for the various PrEP treatment options, which results in a collaborative approach between the provider and patient in developing an individualized treatment plan.

#### Study specific aims

The current study addresses a significant gap in the literature regarding awareness and use of PrEP among Latino MSM in San Antonio, Texas. Existing research has focused on PrEP awareness, but additional knowledge is needed to identify factors associated with PrEP decision-making differences between Latino MSM currently taking PrEP; Latino MSM aware, but not on PrEP; and Latino MSM unaware of PrEP. The current study explores an area that has been overlooked in research: PrEP adherence and side effects, PrEP stigma, and mistrust of the government as well as health care providers. The overall goal of the study is to explore Latino MSMs’ (ages 21–30) beliefs and attitudes associated with PrEP in San Antonio, Texas. The specific aims are to design an instrument that measures PrEP attitudes; identify significant associations between demographic and PrEP related factors; and recommend culturally informed strategies to promote PrEP for Latino MSM.

## Methods

### Sample and procedures

The Institutional Review Board at Texas State University approved research protocols prior to conducting the study. Eligible participants were required to be between the ages of 21 through 30, and identify as a male (gender and biologically) as well as Latino/Hispanic/Spanish. Eligible participants also had to report residence in San Antonio and a sexual encounter (non-specified–i.e., oral, anal) with another male in the past six months. The survey was available in English and Spanish and distributed according to the participants’ preferred language at screening. The entire survey was originally written in English and professionally translated from English to Spanish. A native Spanish speaker that is bilingual back translated the final translation into English.

A total of 636 people (605 in English and 31 in Spanish) attempted to complete the Internet survey, but only 176 individuals (170 in English and 6 in Spanish) were screened eligible for the survey. However, the data analysis for this manuscript will only focus on the English survey (n = 159) due to the low numbers for the Spanish survey (n = 6) and PLWH (n = 5) were excluded for the PrEP portion of the survey. The 30-minute survey was administered anonymously. However, participants had the option to enter their personal information at the end of the survey to receive a $25 Amazon gift card incentive for completing the survey. A total of 155 participants requested the incentive and 4 participants decided to remain anonymous. Personal information of participants was stored in a separate database to protect their privacy and a direct link was not established with the completed survey.

Multiple approaches were used to recruit the target population through convenience-based sampling. The Alamo Area Resource Center (AARC), which is an HIV organization serving the local San Antonio community supported recruitment efforts. Additional members from the Latino MSM community also supported recruitment endeavors and referred eligible participants to the study. Outreach staff distributed study flyers in local gay bars and social venues in San Antonio, Texas. Facebook and gay social networking applications (e.g., Scruff, Grindr) were also used to provide study information and recruit participants. Eligible participants completed a pre-screening questionnaire, consented to participate in the research study, and were offered a $25 Amazon electronic stipend for their participation.

### Measures

The research project sought to explore multiple factors related to HIV transmission. However, this manuscript will focus on Latino MSM attitudes and beliefs related to PrEP (S1 Table & S2 Table). The survey included skipping patterns to explore PrEP attitudes and beliefs among Latino MSM. All participants, with the exception of PLWH, were required to answer questions on PrEP awareness (Q1. Have you ever heard of PrEP/Truvada?) and if they were currently taking PrEP (Q2.2 Are you currently taking PrEP/Truvada?), refer to S1 Table. The question on the source of PrEP information (Q2.1) was only designated for Latino MSM aware of PrEP.

Latino MSM were categorized into three groups based on their responses (S2 Table): 1. Currently taking PrEP (n = 64, 40.3%); 2. Aware, but not on PrEP (n = 37, 23.3%); and 3. Unaware of PrEP (n = 58, 36.5%). Latino MSM currently not on PrEP were provided a brief definition explaining that PrEP/Truvada is a medication that prevents HIV infection. PrEP questions were tailored for Latino MSM currently on PrEP to identify decision-making factors for taking PrEP (S2 Table, left column) and Latino MSM not currently on PrEP to explore decision-making factors for considering PrEP (S2 Table, right column). PrEP adherence placed an emphasis on taking PrEP daily (Q3.2a) and getting an HIV (Q3.2b) as well as blood tests (Q3.2c) every three months. Three questions (Q3.3) explored PrEP associated stigmas for being gay, HIV positive, and promiscuous. Questions 3.4 placed an emphasis on distinguishing PrEP based common side effects (e.g., nausea, dizziness, vomit, diarrhea, stomach pain) in comparison to adverse drug reactions, specifically for concerns related to kidney and liver damage. The distinction for potential drug reactions is important to determine if side effects influence decisions for Latino MSM to consider PrEP, which has not been explored in biomedical interventions for Latino MSM. Only Latino MSM that reported not being on PrEP received questions related to trust in the government (Q4.5–2 questions) and PrEP issues related to health care providers (Q4.6–2 questions).

SPSS software version 24 was used to conduct a descriptive and bivariate analysis of the data [46]. Research participants for the quantitative survey (S2 Table) were stratified to compare and contrast issues related to PrEP between the designated groups (1. Currently Taking PrEP; 2. Aware, but not on PrEP; and 3. Unaware of PrEP). In addition, Likert scale responses were collapsed into one category in order to compare the three different groups for similar questions (S2 Table sections for Q3.2 and Q3.4) with dichotomous responses for yes (i.e., strongly agree to agree) and no (i.e., disagree to strongly disagree).

## Results

Descriptive demographic information for participants is provided in S3 Table. Participant mean age was 25.09 (SD 2.05). Eligibility criteria required that all participants have at least one sexual encounter with a male within the past six months. PrEP awareness overall was significantly associated with age, education, and annual household income. Participants that reported to be younger were less likely to know about PrEP (r (159) = .163, p < .05). Lower educational attainment was also significantly associated with lower levels of PrEP awareness (r (159) = .517, p < .001). Latino MSM with lower levels of reported annual household income was significantly associated with lower levels of PrEP awareness (r (159) = .647, p < .001). Latino MSM that reported currently taking PrEP was significantly associated with greater levels of education (r (159) = .510, p < .001) and household annual income (r (159) = .706, p < .001).

### PrEP awareness & decision-making

**Fig 1:**
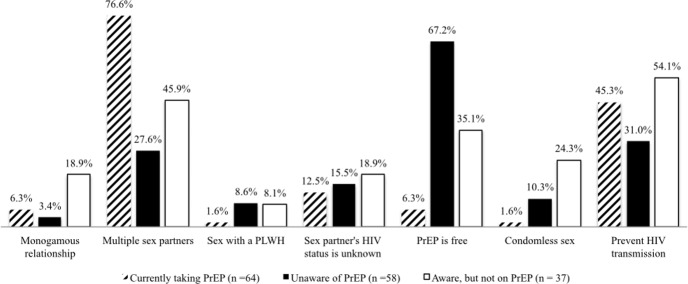
Latino MSM (N = 159) decision-making factors for taking (currently on PrEP) or considering PrEP (not currently on PrEP).

Latino MSM aware of PrEP (n = 101) reported that the primary source of PrEP information was through the Internet (39%) followed by a sex partner (37.1%), friend (29.6%), and/or health care professional (25.8%). Only 40% of the participants reported they are currently on PrEP among Latino MSM aware of PrEP. Fig 1 provides a comparison between the groups of Latino MSM on decision-making factors to take (Currently on PrEP) or consider taking PrEP (Aware, but not on PrEP; Unaware of PrEP). The primary reasons Latino MSM reported taking PrEP were because they had multiple sex partners (76.6%) and they did not want to get infected with HIV (45.3%). Latino MSM not on PrEP (n = 95) reported that HIV prevention (40%) and having multiple sex partners (34.7%) were important factors for consideration in their PrEP decision-making. Previous research has indicated that increased condomless sex would occur if individuals were on PrEP [10], but only 1.6% of participants on PrEP and 15.8% of participants not on PrEP reported that condomless sex is an incentive to take PrEP. However, Latino MSM aware of PrEP and not currently on PrEP were more likely (24.3%) to report not using condoms is an important reason to consider PrEP.

### PrEP associated stigmas

**Fig 2:**
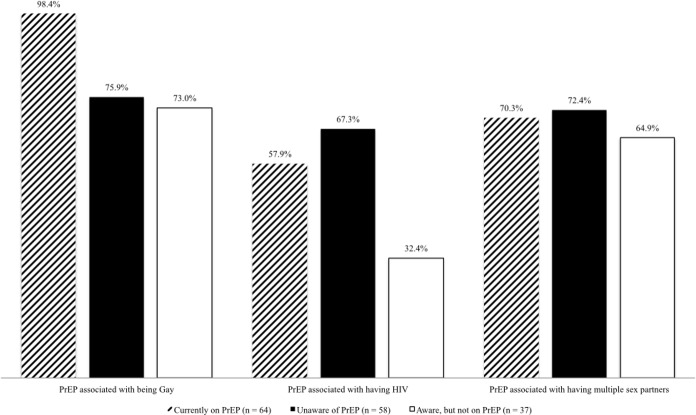
PrEP associated stigmas for Latino MSM (N = 159).

Fig 2 provides information on PrEP associated stigmas reported by Latino MSM. A majority of Latino MSM reported that taking PrEP would cause people to stigmatize them as gay (98.4% on PrEP and 74.8% not on PrEP), HIV positive (57.9% on PrEP and 53.7% not on PrEP), and promiscuous (70.3% on PrEP and 69.5% not on PrEP). Latino MSM unaware of PrEP reported higher levels of being stigmatized for taking PrEP as being a PLWH (67.3%) and promiscuous (72.4%).

### PrEP associated side effects

**Fig 3:**
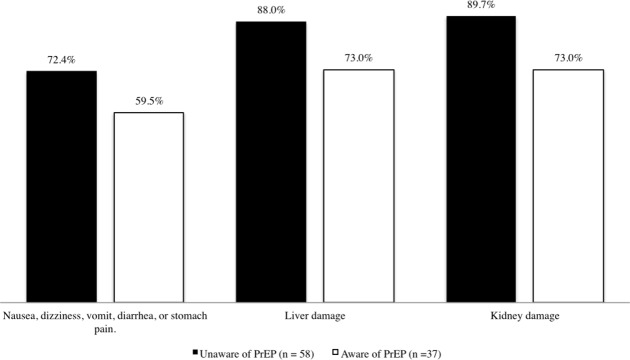
Latino MSM (not currently on PrEP) unwilling to take PrEP due to potential side effects.

Latino MSM currently on PrEP reported that they received information on potential side effects such as nausea, dizziness, vomit, diarrhea, or stomach pain (95.3%); liver damage (81.3%); and kidney damage (84.4%) by their health care provider prior to beginning PrEP. However, Latino MSM not currently on PrEP reported that they are less likely to consider PrEP due to potential side effects such as nausea, dizziness, vomit, diarrhea, or stomach pain (67.4%); liver damage (82.1%); and kidney damage (83.1%). Latino MSM that never heard of PrEP had the greatest concerns for unwillingness to consider PrEP due to potential side effects. PrEP common side effects and adverse drug reactions reported by Latino MSM not currently on PrEP are specified in Fig 3.

### Trust in the government & issues related to medical providers

**Fig 4:**
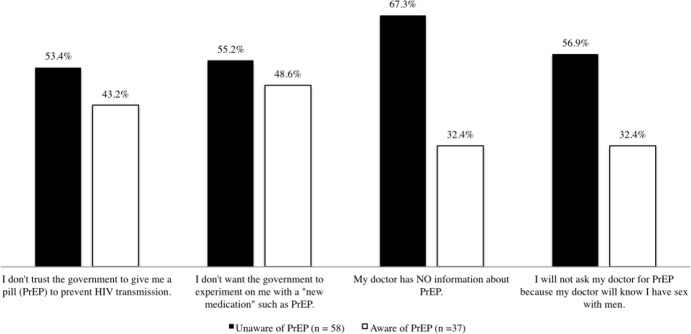
Latino MSM (not currently on PrEP) perceptions associated with the government and medical provider regarding PrEP.

Data from Latino MSM not currently on PrEP demonstrated mistrust in the government (Fig 4). Questions related to mistrust in the government was limited to Latino MSM not currently on PrEP. Participants were divided (50.6%) and reported that they do not trust the government giving them a pill to prevent HIV transmission and 47.4% reported that they do not want the government to experiment with them taking a new medication such as PrEP/Truvada. Latino MSM that never heard of PrEP had higher levels of mistrusting the government when compared to Latino MSM that had heard of PrEP and currently not on PrEP.

Slightly more than half of Latino MSM participants not currently on PrEP (53.7%) reported that their doctor did not have information about PrEP. Less than half (47.4%) reported that they would not ask their doctor for PrEP because they did not want to disclose that they engaged in sex with men. However, Latino MSM unaware of PrEP reported at higher rates that their doctor had no information related to PrEP (67.3%) and 56.9% reported that they were more concerned that their doctor would discover that they engaged in sexual activity with other men.

### PrEP adherence

**Fig 5:**
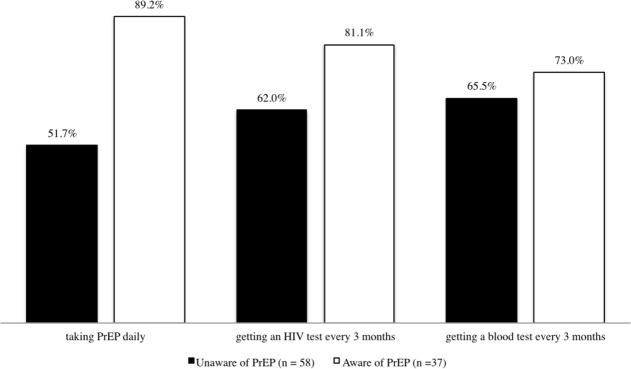
Latino MSM (not currently on PrEP) willing to take PrEP due to the required treatment regimen for taking PrEP daily, getting an HIV and blood tests every 3 months.

Overall PrEP adherence is low among current users, only 46.9% reported that they took PrEP daily, 10.9% reported that they had an HIV test every three months, and 25% reported that they had laboratory testing every three months. Latino MSM currently not on PrEP would be willing to take a pill daily (66.3%). Among this group 69.4% would be willing to have an HIV test every three months, and 68.4% would obtain laboratory testing every three months (Fig 5). However, Latino MSM aware of PrEP but not currently taking PrEP reported greater rates of unwillingness to take a pill daily (89.2%), take an HIV test every three months (81.1%), or have laboratory tests every three months (73%).

## Discussion

The results of this study provide critical insight on PrEP as a biomedical intervention for Latino MSM. The data from this study emphasizes PrEP related issues that have been overlooked in prior studies on PrEP, especially among Latino MSM in the South. In order to understand PrEP decision-making factors for Latino MSM consideration must be placed on distinguishing participants among the Latino MSM population including: 1) current PrEP users; 2) individuals aware of, but not on PrEP; and 3) individuals unaware of PrEP. These distinctions are important as they identify factors that motivate or discourage Latino MSM who might be willing to consider PrEP or those who are currently on PrEP. Structural factors identified in this study for Latino MSM include: access to free PrEP medication, insurance coverage issues, adherence to follow-up medical visits, PrEP related stigmas, access to PrEP knowledgeable health care providers, and mistrust of the government as well as health care providers. Individual factors associated with Latino MSM are preconceived notions regarding PrEP effects and their willingness to consider PrEP as a viable option to prevent HIV transmission, especially in regards to PrEP adherence and potential adverse reactions.

One primary reason for PrEP use reported by participants who were currently on PrEP was their continued engagement with multiple sexual partners. Prior research has demonstrated that current PrEP users are aware of their risks and have taken preventive measures to reduce HIV transmission [47]. Our analysis demonstrated that household income and educational level was an important factor in PrEP awareness. Participants with lower income levels and less education were more likely to be unaware of PrEP. Therefore, PrEP educational programs must target lower socioeconomic level groups as well as those with lower educational attainment. Latino MSM unaware of PrEP have expressed concerns about the cost of PrEP, which emphasizes the need to provide free or low cost PrEP for these groups.

Surprisingly, only 31% of Latino MSM unaware of PrEP reported that HIV prevention was an important factor in considering PrEP. These results require further investigation as previous research found a preference for condom use rather than PrEP among MSM that report lower educational attainment [47]. Latino MSM for this study overall did not report condomless sex as the top three reasons to take or consider PrEP. However, approximately 25% of Latino MSM aware, but not on PrEP in this study ranked the highest for selecting condomless sex as an incentive to take PrEP.

The majority of Latino MSM reported PrEP associated stigmas related to being gay, PLWH, and promiscuous. Special consideration should be placed on PrEP promotion strategies for Latino MSM unaware of PrEP. Overall, 75.9% of Latino MSM unaware of PrEP reported that PrEP is associated with being gay and 56.9% indicated that they would not ask a doctor for PrEP to avoid disclosing MSM related behavior. These data emphasize the importance of developing culturally appropriate PrEP educational and promotional programs that include individual and structural level factors that influence decision-making among Latino MSM. Especially in regards to biomedical interventions addressing PrEP associated stigmas of being promiscuous or a PLWH.

Factors associated with mistrust of the government and medical providers among MSM of color [48–50] create additional barriers for government and state initiatives supporting PrEP as a biomedical intervention for high-risk populations such as MSM of color. Latino MSM in this study indicated that mistrust of the government and medical community highly influenced their decision-making around PrEP. A significant number reported mistrust of the government and medication programs that target the MSM population in particular. Distrust of the government and medical providers is not new, there have been multiple studies in which participants site mistrust as a reason for not seeking care [48–50]. A recent study which examined PrEP information on Twitter found that negative tweets about PrEP were more likely to be replicated than tweets with positive information [51]. In addition prior research has found that MSM living in a highly stigmatizing environment were less likely to be knowledgeable and have access to biomedical HIV prevention strategies and more likely to engage in risky sexual behavior [52]. This suggests that negative information about PrEP including medical mistrust and conspiracy theories about its use may play a role in patients’ decision-making. For example, HIV positive Latino men (N = 208–61% Gay, 15% Bisexual, 22% Straight) reported that perceived discrimination based on being Latino and/or HIV positive was significantly associated with lower levels of HIV medication adherence [22].

In addition to mistrust of the government and medical community, stigma may play a role in awareness of and access to PrEP. Prior research among Black MSM in the South found stigmatizing attitudes, lower levels of PrEP awareness, access to PrEP, and acceptance of PrEP for MSM of color by health care providers and the general population [39, 40]. Tailored PrEP advertising for high-risk populations may inadvertently stigmatize MSM of color and reinforce barriers for access to PrEP. Therefore, it is important that PrEP education and promotion programs target the community at large [40]. In addition to community programs additional PrEP education and training of health care providers is imperative. Emphasis on basic HIV knowledge, risk assessment, and routine HIV testing as well as PrEP patient education and administration protocols within a multidisciplinary patient-centered approach is necessary [39, 43]. Furthermore, programs must take into consideration issues of mistrust of the government and the medical community when developing and implementing PrEP interventions.

There are many misconceptions and preconceived beliefs associated with PrEP. For instance, many young MSM (43% Latino) expressed that PrEP may be more appropriate for men with multiple sex partners, substance users, and serodiscordant couples [9]. Initiatives taken by gay social applications (e.g., Adam4Adam, Grindr, Scruff) for MSM to disclose their HIV and PrEP status are important to raise awareness, but it also may reinforce the stigma of “PrEP/Truvada whores” referring to MSM that want to have condomless sex with a lot of men. MSM report that taking PrEP is associated with the intent to have sex daily [9]. MSM that had lower levels of PrEP stigma were more likely to accept PrEP as an intervention [11]. Marketing PrEP for high-risk populations inadvertently stigmatize MSM on PrEP, which may discourage potential candidates from considering PrEP as an effective method to prevent HIV transmission. Latino MSM participants in this study reported that people would stigmatize PrEP users as being gay, sexually promiscuous, and as a PLWH.

In addition to the general individual and structural level barriers to PrEP there may be other barriers to consider for the Latino population such as immigration status, language, and access to care. PrEP programs must take into consideration legal and governmental policies when developing culturally informed interventions for Latino MSM in highly conservative areas such as the South. Latino MSM in San Antonio expressed concerns related to participating in this study regarding privacy and confidentiality issues. However, outreach workers from the Latino MSM community played a critical role in gaining the trust of the Latino MSM community and encouraged them to support research endeavors. Culturally informed PrEP promotion and messaging should reinforce the role of Latino MSM in the community. Research has demonstrated that MSM peer testimonials about PrEP have been an effective approach for community education on HIV transmission [44]. Therefore, recruiting MSM PrEP users to become peer PrEP educators, who are culturally informed and able to integrate cultural beliefs is an effective approach for addressing community concerns related to HIV/AIDS and PrEP. MSM peer educators are able to share their personal experiences using PrEP as well as explain research findings and address issues of medical distrust among MSM of color [16, 20, 21]. Employing an interdisciplinary approach in developing biomedical interventions for diverse populations is supported by research as an effective tool [9]. However, additional strategies need to be employed to raise awareness of PrEP within the MSM community as well as social service and health care providers.

Researchers emphasize the need to further understand approaches for MSM to access information and improve PrEP linkage to care [53]. Individual decision-making factors such as PrEP adherence are important to consider for Latino MSM. Less than half of Latino MSM who were currently on PrEP reported not taking the medication daily. However, the survey did not inquire if Latino MSM took PrEP at least four times a week and blood samples were not collected at the time of the study to determine the medication possession ratio for PrEP adherence [29]. Additional factors associated with substance abuse and/or pre-existing mental health conditions was not explored to determine motivation for PrEP adherence [31]. However, findings for this study stress the importance of supplementing biomedical interventions with patient-centered cognitive behavioral therapy approaches [30]. Also, willingness to take PrEP for Latino MSM provides critical information on identifying potential barriers to accept PrEP as an intervention. Findings for this study corroborate prior research [8, 9] that taking a daily pill is the primary reason (89.2%) not to take PrEP among Latino MSM aware, but currently not on PrEP. A barrier for taking PrEP daily emphasizes the need to provide patients with multiple options tailored to their needs and consider various approaches for administering PrEP such as “on-demand” [32], long-acting PrEP injections [16], and short-term PrEP regimens during high-risk periods [35, 36]. Overall, motivational incentives (e.g., STI testing, mental health services, substance use services) should be emphasized when encouraging PrEP medication initiation and adherence.

In addition to PrEP adherence, professionals must address concerns related to PrEP side effects and the importance of follow-up medical visits. Researchers have explored factors effecting PrEP use and general side effects among MSM [8–11, 13–19]. However, a distinction must be made between common side effects and severe adverse reactions to determine participants’ willingness to consider PrEP. Currently, there are no studies that have explored willingness to take PrEP based on the type of possible PrEP related side effects. This is an important distinction as many Latino MSM weigh the possibility of dealing with common side effects verses severe adverse reactions. Latino MSM unaware of PrEP were approximately three times less likely to consider PrEP due to common side effects and over 88% reported that a potential adverse side effect makes it unlikely that they will consider PrEP. PrEP interventions targeting Latino MSM must address the issue of side effects and adverse reactions.

This study explored awareness and attitudes about PrEP among a sample of Latino MSM. However, there are some limitations to this study. Participants in this community reported a higher educational and income level than the Latino population in general [54, 55]. This might have skewed the data as participants with higher incomes and educational levels may be more aware of and willing to take PrEP. Additionally, all of the data collection was self-reported. Self-administered surveys online required access to a computer and the Internet, which may not be available in low-income households. However, the survey was also available through smart phones to strengthen accessibility for Latino MSM. The research study was available in English and Spanish, but the study encountered limited access to Spanish speaking Latino MSM and only 6 surveys were completed in Spanish. Promotional materials (e.g., flyers, social media) were available in English and Spanish. However, the geographical location of the research in San Antonio, Texas may have also raised concerns for participating in a research study that explored highly sensitive issues (e.g., sexuality, substance use, HIV status) within a state that is considered socially and politically conservative. For these reasons individuals may have been discouraged to participate out of fear of being stigmatized as gay or as a PLWH. Immigration status may have been an additional barrier, discouraging highly marginalized Latino MSM to participate in a research study. The survey also did not include questions about mistrust of the government and health care providers for Latino MSM currently on PrEP. Future studies should include questions about government and medical provider mistrust issues for all participants. PLWH were also excluded from completing the PrEP survey, but a study indicated that PLWH play a critical role on recommending PrEP to their sexual partner [56]. Therefore, further research on PrEP awareness is needed as well as potential roles PLWH are able to provide for promoting PrEP as a biomedical intervention for high-risk populations. Lastly, the results of this research are not generalizable to all Latino MSM due to the location and sample size. However, despite limitations the study enhances our understanding of PrEP as a biomedical intervention with Latino MSM.

## Conclusion

The PrEP instrument developed for this study provided critical information to further understand PrEP related attitudes and decision-making factors for Latino MSM. Overall, the findings for individual factors emphasize the need for patient-centered interventions for Latino MSM. Latino MSM currently on PrEP require supplemental resources to enhance PrEP adherence. Latino MSM not on PrEP require alternate options for PrEP delivery and/or cognitive behavioral approaches minimizing HIV risk behavior for Latino MSM concerned with PrEP toxicity, which may require non-biomedical interventions.

Overall, biomedical PrEP interventions for Latino MSM must address issues related to mistrust of the government and medical providers. Peer educators currently on PrEP from the local Latino MSM community may be a viable option in overcoming obstacles related to mistrust with the government and medical providers. Latino MSM peer educators ideally should have established social networks to reinforce their influence on raising PrEP awareness and contextualizing PrEP related concerns based on their personal experience. PLWH should also not be overlooked and integrated in the promotion of PrEP with the Latino MSM community. Empowering the community to become active members in initiating HIV awareness and biomedical PrEP interventions reinforces a culturally informed approach that allows individuals the ability to relate to peers who exemplify the benefits of PrEP. Additionally, an interdisciplinary perspective is critical for taking a holistic approach in tailoring patient-centered PrEP interventions addressing biological, psychological, social, and structural HIV related risk factors for Latino MSM.

## Supporting information

S1 Table Quantitative survey PrEP awareness questions and participant responses (N = 159). (PDF)

S2 Table Quantitative survey PrEP questions and participant responses (N = 159) designated by representative groups for Latino MSM. (PDF)

S3 Table Descriptive characteristics of PrEP participants for self-administered internet surveys (N = 159). (PDF)
