# Supplementary material for: PrEP awareness and decision-making for Latino MSM in San Antonio, Texas
Source: PLoS One. 2017 Sep 27;12(9):e0184014. doi: 10.1371/journal.pone.0184014 (PMC5617149; doi:10.1371/journal.pone.0184014)
Supplement: S1 Table — (PDF) [file pone.0184014.s001.pdf]

S1 Table. Quantitative survey PrEP awareness questions and participant responses (N = 159).

Question for All Participants	*n*	*(%)*
Q1. Have you ever heard of PrEP/Truvada?		
Yes	101	(63.5)
No	58	(36.5)
Questions for Participants that have heard of PrEP (N = 101)		
Q2.1. Please select all of the following options that provided you with information about PrEP/Truvada: (Check all that apply)		
Healthcare professional (doctor, nurse, etc.)	41	(25.8)
Someone at the health department	27	(17)
Outreach worker	6	(3.8)
Sex partner	59	(37.1)
Drug sharing partner	6	(3.8)
Friend	47	(29.6)
Family member	11	(6.9)
Media/advertisement	16	(10.1)
Internet	62	(39)
Someone else, specify:	1	(0.6)
Q2.2. Are you currently taking PrEP/Truvada?		
Yes	64	(40.3)
No	37	(23.3)
