# Supplementary material for: PrEP awareness and decision-making for Latino MSM in San Antonio, Texas
Source: PLoS One. 2017 Sep 27;12(9):e0184014. doi: 10.1371/journal.pone.0184014 (PMC5617149; doi:10.1371/journal.pone.0184014)
Supplement: S2 Table — (PDF) [file pone.0184014.s002.pdf]

S2 Table. Quantitative survey PrEP questions and participant responses (N = 159) designated by representative groups for Latino MSM.

Questions for Participants…	Currently on PrEP		Currently not on PrEP		Currently not on PrEP Groups			
					Unaware of PrEP		Aware of PrEP	
	n	*(%)*	*n*	*(%)*	*n*	*(%)*	*n*	*(%)*
Q3.1. Why did you decide to take PrEP/Truvada? (Check all that apply)			Would you be willing to take PrEP/Truvada (the medication/drug that prevents HIV transmission) because…(Check all that apply)					
I am in a monogamous relationship with someone that has HIV.	4	(6.3)	9	(9.5)	2	(3.4)	7	(18.9)
I have multiple sex partners.	49	(76.6)	33	(34.7)	16	(27.6)	17	(45.9)
I am having sex with someone that has HIV.	1	(1.6)	8	(8.4)	5	(8.6)	3	(8.1)
I do not know if my sex partners have HIV.	8	(12.5)	16	(16.8)	9	(15.5)	7	(18.9)
PrEP/Truvada is free.	4	(6.3)	16	(16.8)	39	(67.2)	13	(35.1)
I do NOT want to use condoms.	1	(1.6)	15	(15.8)	6	(10.3)	9	(24.3)
I do NOT want to get infected with HIV.	29	(45.3)	38	(40)	18	(31)	20	(54.1)
Q3.2a. Have you taken PrEP/Truvada EVERY DAY?			Would you be willing to take PrEP/Truvada EVERY DAY?					
* Yes	30	(46.9)	63	(66.3)	30	(51.7)	33	(89.2)
* No	34	(53.1)	32	(33.7)	28	(48.3)	4	(10.8)
Q3.2b. Do you get an HIV test every 3 months?			Would you be willing to get an HIV test every 3 months?					
* Yes	7	(10.9)	66	(69.4)	36	(62)	30	(81.1)
* No	57	(89.1)	29	(30.5)	22	(37.9)	7	(18.9)
Q3.2c. Do you get a blood test every 3 months?			Would you be willing to get a blood test every 3 months?					
* Yes	16	(25)	65	(68.4)	38	(65.5)	27	(73)
* No	48	(75)	30	(31.6)	20	(34.4)	10	(27)
Q3.3. Taking PrEP/Truvada causes people to think that...			Taking PrEP/Truvada MIGHT cause people to think that…					
I'm gay.			I'm gay.					
Strongly Disagree	0		4	(4.2)	2	(3.4)	2	(5.4)
Disagree	1	(1.6)	20	(21.1)	12	(20.7)	8	(21.6)
Agree	55	(85.9)	66	(69.5)	41	(70.7)	25	(67.6)
Strongly Agree	8	(12.5)	5	(5.3)	3	(5.2)	2	(5.4)
I have HIV.			I have HIV.					
Strongly Disagree	13	(20.3)	9	(9.5)	5	(8.6)	4	(10.8)
Disagree	14	(21.9)	35	(36.8)	14	(24.1)	21	(56.8)
Agree	36	(56.3)	46	(48.4)	36	(62.1)	10	(27)
Strongly Agree	1	(1.6)	5	(5.3)	3	(5.2)	2	(5.4)
I have sex with a lot of guys.			I have sex with a lot of guys.					
Strongly Disagree	8	(12.5)	9	(9.5)	7	(12.1)	2	(5.4)
Disagree	11	(17.2)	20	(21.1)	9	(15.5)	11	(29.7)
Agree	39	(60.9)	59	(62.1)	38	(65.5)	21	(56.8)
Strongly Agree	6	(9.4)	7	(7.4)	4	(6.9)	3	(8.1)
Q3.4. Did your doctor explain to you that PrEP/Truvada might cause the following symptoms ...			Would you be willing to take PrEP/Truvada if it might cause the following symptoms...					
Nausea, dizziness, vomit, diarrhea, or stomach pain.			Nausea, dizziness, vomit, diarrhea, or stomach pain.					
* Yes	61	(95.3)	31	(32.7)	16	(27.6)	15	(40.5)
* No	3	(4.7)	64	(67.4)	42	(72.4)	22	(59.5)
Liver damage			Liver damage					
* Yes	52	(81.3)	17	(17.9)	7	(12)	10	(27)

* No	12	(18.7)		78	(82.1)	51	(88)	27	(73)
Kidney damage			Kidney damage						
* Yes	54	(84.4)		16	(16.9)	6	(10.3)	10	(27)
* No	10	(15.6)		79	(72.1)	52	(89.7)	27	(73)
Q4.5. Trust in Government Questions			Trust in Government Questions						
I don't trust the government giving me a pill (PrEP/Truvada) to prevent HIV transmission.			I don't trust the government giving me a pill (PrEP/Truvada) to prevent HIV transmission.						
Strongly Disagree	NA			5	(5.3)	3	(5.2)	2	(5.4)
Disagree	NA			43	(45.3)	24	(41.4)	19	(51.4)
Agree	NA			40	(42.1)	26	(44.8)	14	(37.8)
Strongly Agree	NA			7	(7.4)	5	(8.6)	2	(5.4)
I don't want the government to experiment with me taking a new medication (PrEP/Truvada).			I don't want the government to experiment with me taking a new medication (PrEP/Truvada).						
Strongly Disagree	NA			6	(6.3)	4	(6.9)	2	(5.4)
Disagree	NA			39	(41.1)	22	(37.9)	17	(45.9)
Agree	NA			44	(46.3)	28	(48.3)	16	(43.2)
Strongly Agree	NA			6	(6.3)	4	(6.9)	2	(5.4)
Q4.6. Medical Provider			Medical Provider						
My doctor doesn't have information about PrEP/Truvada.			My doctor doesn't have information about PrEP/Truvada.						
Strongly Disagree	NA			6	(6.3)	3	(5.2)	3	(8.1)
Disagree	NA			38	(40.0)	16	(27.6)	22	(59.5)
Agree	NA			46	(48.4)	36	(62.1)	10	(27)
Strongly Agree	NA			5	(5.3)	3	(5.2)	2	(5.4)
I don't ask my doctor for PrEP/Truvada because my doctor will know I have sex with guys.			I don't ask my doctor for PrEP/Truvada because my doctor will know I have sex with guys.						
Strongly Disagree	NA			8	(8.4)	3	(5.2)	5	(13.5)
Disagree	NA			42	(44.2)	22	(37.9)	20	(54.1)
Agree	NA			38	(40.0)	30	(51.7)	8	(21.6)
Strongly Agree	NA			7	(7.4)	3	(5.2)	4	(10.8)

* Indicates that Likert scale responses were collapsed into one category for Latino MSM not on PrEP in order to compare with Latino MSM currently on PrEP for similar questions with dichotomous responses for yes (i.e., strongly agree to agree) and no (i.e., disagree to strongly disagree). NA indicates that the participants' did not receive the question.
