# Supplementary material for: PrEP awareness and decision-making for Latino MSM in San Antonio, Texas
Source: PLoS One. 2017 Sep 27;12(9):e0184014. doi: 10.1371/journal.pone.0184014 (PMC5617149; doi:10.1371/journal.pone.0184014)
Supplement: S3 Table — (PDF) [file pone.0184014.s003.pdf]

S3 Table. Descriptive characteristics of PrEP participants for self-administered Internet surveys (N = 159).		
**Characteristics**	***n***	***(%)***
**Race**		
Latino/Spanish/Hispanic	154	(96.9)
White & Latino/Spanish/Hispanic	4	(2.5)
Black & Latino/Spanish/Hispanic	1	(0.6)
**Ethnicity**		
Mexican	147	(92.5)
Puerto Rican	6	(3.8)
Cuban	4	(2.5)
Spanish	2	(1.3)
Colombian	1	(0.6)
Venezuelan	1	(0.6)
**Country of Birth**		
United States	147	(92.5)
Cuba	4	(2.5)
Mexico	5	(3.1)
Colombia	1	(0.6)
Venezuela	1	(0.6)
**Education**		
Grade 1-11	0	
Grade 12 - No Diploma	1	(0.6)
Grade 12 - With Diploma	23	(14.5)
GED or alternative credential	2	(1.3)
Some college credit	36	(22.6)
Associate's Degree	9	(5.7)
Bachelor's Degree	76	(47.8)
Master's Degree	9	(5.7)
Professional degree beyond a Bachelor's Degree (for example: MD, DDS, DVM, JD)	2	(1.3)
Doctorate Degree	1	(0.6)
**Annual Household Income**		
Less than 9,999	26	(16.4)
10,000 - 19,999	23	(14.5)
20,000 - 29,999	15	(9.4)
30,000 - 39,999	7	(4.4)
40,000 - 49,999	6	(3.8)
50,000 - 59,999	12	(7.5)
60,000 - 69,999	12	(7.5)
70,000 or more	58	(36.5)
**Marital Status**		

Single/never married	151	(95)
Married	6	(3.8)
Divorced	1	(0.6)
Separated	1	(0.6)
**Sexual Identity**		
Gay	144	(90.6)
Bisexual	13	(8.2)
Straight	1	(0.6)
Other: "I get down with whatever"	1	(0.6)
**Sex with a Female past 6 months**	67	(42.1)
